# Recent Advances in Targeting the EGFR Signaling Pathway for the Treatment of Metastatic Colorectal Cancer

**DOI:** 10.3390/ijms18040752

**Published:** 2017-04-02

**Authors:** Yuji Miyamoto, Koichi Suyama, Hideo Baba

**Affiliations:** 1 Department of Gastroenterological Surgery, Graduate School of Medical Sciences, Kumamoto University, 1-1-1 Honjo, Kumamoto 860-8556, Japan; miyamotoyuji@gmail.com; 2 Cancer Center, Kumamoto University Hospital, Kumamoto 860-8556, Japan; kou_susan@yahoo.co.jp

**Keywords:** metastatic colorectal cancer, epidermal growth-factor receptor signaling (EGFR), chemotherapy, secondary resistance

## Abstract

Outcomes for metastatic colorectal cancer (mCRC) patients have been improved by treatment with anti-epidermal growth factor receptor (anti-EGFR) antibodies, particularly when combined with predictive biomarkers to select patients lacking *RAS* mutations. New technologies such as liquid biopsy and next-generation sequencing have revealed that potential mechanisms of resistance to anti-EGFR therapies act through acquired mutations of *KRAS* and the *EGFR* ectodomain. Mutations in cross-talking molecular effectors that participate in downstream EGFR signaling are also negative predictors for anti-EGFR therapy. In the current review, we describe recent advances in anti-EGFR therapy and discuss new treatment strategies to target downstream RAS-MAPK signaling in mCRC.

## 1. Introduction

In recent decades, significant improvements have been seen in the survival of patients with metastatic colorectal cancer (mCRC). This has been driven to a large extent by the approval of new drugs, including irinotecan, oxaliplatin, capecitabine, several humanized monoclonal antibodies that target either vascular endothelial growth factor (VEGF; bevacizumab, aflibercept, and ramucirumab) or the epidermal growth factor receptor (EGFR; cetuximab and panitumumab), and most recently, regorafenib and trifluridine/tipiracil (TAS-102) [1,2,3,4,5,6,7,8,9,10,11,12,13]. Clinical benefit from these drugs is now well established for patients with mCRC, with median overall survival (OS) of over 30 months (M) [14,15,16]. In addition, a number of studies have focused on the identification of more reliable and specific predictive biomarkers for choosing the most appropriate treatment for an individual patient. The only established biomarker for the treatment of mCRC patients is tumor *RAS* mutational status, which is a negative predictive marker for anti-EGFR therapy [17]. Currently, *BRAF* mutational testing is also recommended by the National Comprehensive Cancer Network (NCCN) [18] and the European Society for Medical Oncology (ESMO) [19].

Unlike anti-VEGF therapy, the mechanisms of resistance in anti-EGFR therapy are well-studied, as are drugs inhibiting downstream RAS-MAPK signaling. Indeed, several recent clinical trials targeting RAS signaling have shown promising activity in chemorefractory mCRC. In this review, we focus on recent clinical and preclinical studies of EGFR inhibitors, their resistance mechanisms, and new downstream inhibitors of the EGFR pathway.

## 2. Clinical Advances in Anti-EGFR Antibodies

Cetuximab and panitumumab are both monoclonal antibodies directed against the extracellular domain of the EGFR, which block ligand binding and lead to inhibition of the downstream RAS-RAF-MEK-ERK signaling pathway. Several randomized clinical trials have established the effectiveness of both drugs in combination with fluorouracil (5-FU) plus irinotecan (FOLFIRI) [8,20] and 5-FU plus oxaliplatin (FOLFOX) [10,21] for patients with wild-type *RAS* mCRC. The differences between cetuximab and panitumumab might be derived from their different protein class characteristics or species (cetuximab is 13% mouse and 87% human, panitumumab is 100% human) [22]. As an IgG1 antibody, cetuximab exerts additional antitumor effects by mediating antibody-dependent cellular cytotoxicity [23]. However, the significance of this effect is not fully understood. The ASPECCT study [24], a phase III randomized controlled trial, indicated that panitumumab was non-inferior to cetuximab and that these agents provided a similar OS benefit in patients with chemotherapy-refractory wild-type *KRAS* exon 2 mCRC. The primary endpoint, median OS was 10.4 M in the panitumumab group and 10.0 M in the cetuximab group (hazard ratio, HR = 0.97, 95% confidence interval, CI = 0.84–1.11, *p* < 0.0007 for non-inferiority). These results also showed that the incidence of grade 3 or 4 hypomagnesemia was greater in patients receiving panitumumab (7%) than in those receiving cetuximab (3%), although the incidence of severe skin toxicities was similar in the two groups.

## 3. The Effect of RAS Status on Anti-EGFR Therapies

The search for a predictive biomarker for anti-EGFR therapies was initially directed toward EGFR expression, which has been reported to be increased in 49% to 82% of mCRC [25,26]. Anti-EGFR therapies inhibit downstream signaling pathways, but *EGFR* expression status, as assessed using immunohistochemistry, does not predict treatment efficacy [27,28]. Data from the BOND study indicated that the intensity of immunohistochemical EGFR staining in colorectal tumor cells did not correlate with the objective response rate (ORR) to cetuximab [29]. Subsequently, mutations conferring resistance to anti-EGFR therapies were identified in codons 12 and 13 of exon 2 of the *KRAS* gene, which result in constitutive activation of the RAS-RAF-MEK-ERK pathway [8,21,30,31]. Activating mutations in *KRAS* are detected in approximately 40% of mCRC [31], with good concordance between the primary tumors and matched distant metastases [32,33].

More recent studies have found that resistance to anti-EGFR therapy can also be mediated by lower-frequency mutations in *KRAS* exon 3 or 4, or in *NRAS* exon 2, 3, or 4 [11,32,34]. Exclusion of patients with any *RAS* mutation identifies a population that is more likely to benefit from anti-EGFR therapies [35]. In the PRIME trial [33], 17% of patients without mutations in *KRAS* exon 2 did have mutations in *KRAS* exon 3 or 4, or in *NRAS* exon 2, 3, or 4. All of these *RAS* mutations predicted a lack of response to panitumumab, and in fact, their presence was associated with inferior progression-free survival (PFS) and OS in patients receiving panitumumab plus FOLFOX compared with FOLFOX alone. Median OS was 25.8 M versus 20.2 M (HR = 0.77, 95% CI = 0.64–0.94, *p* = 0.009) in wild-type *RAS* populations, in favor of the combination of panitumumab and FOLFOX. Similar results were presented for all *RAS* genotypes in the CRYSTAL [36] and OPUS [37] trials, in which randomized patients received first-line cetuximab in combination with FOLFIRI or FOLFOX respectively [38]. In addition, a meta-analysis of nine randomized controlled trials of anti-EGFR antibodies for mCRC demonstrated the predictive value of *RAS* mutational profiles for both PFS and OS [35]. These results indicate that the anti-EGFR antibodies should be restricted to mCRC patients whose tumors lack all *KRAS* and *NRAS* mutations.

## 4. *KRAS*^G13D^ Mutation

Preclinical studies and retrospective data from phase III trials suggest that patients with *KRAS^G13D^* mutant tumors might benefit from cetuximab. De Roock et al. reported that patients carrying the *KRAS^G13D^* mutation and treated with cetuximab had prolonged OS (HR = 0.50, 95% CI = 0.31–0.81, *p* = 0.005) and PFS (HR = 0.51, 95% CI = 0.32–0.81, *p* = 0.004) compared with patients whose tumors harbored other *KRAS* mutations [39]. Another retrospective analysis was performed on a pooled data set from the CRYSTAL and OPUS studies [40]. Within *KRAS* mutation subgroups, cetuximab plus chemotherapy versus chemotherapy alone significantly improved PFS (median, 7.4 versus 6.0 M, HR = 0.47, *p* = 0.039) and tumor response rate (41% versus 22%, odds ratio, OR = 3.38, *p* = 0.042) in patients with *KRAS^G13D^* mutant tumors. Based on these results, two prospective phase II trials were performed; however, both trials showed no activity for cetuximab monotherapy in patients with *KRAS^G13D^* mutant mCRC. In the phase II ICECREAM trial [41], there was no statistically significant improvement in disease control at 6 M for patients with *KRAS^G13D^* mutant tumors treated with either cetuximab monotherapy or cetuximab plus irinotecan. Currently, anti-EGFR therapies are not routinely recommended for patients with *KRAS^G13D^* mutant tumors.

## 5. Anti-EGFR Therapies versus Bevacizumab in First Line Chemotherapy

A highly important unresolved issue is whether the anti-VEGF bevacizumab or an anti-EGFR antibody is the optimal first-line biologic agent to be added to chemotherapy for patients with wild-type *RAS* mCRC. Two phase III trials, FIRE-3 [14] and CALGB/SWOG 80405 [42], and the phase II PEAK trial [43] have directly compared the addition of bevacizumab versus cetuximab or panitumumab to FOLFOX/FOLFIRI in terms of efficacy outcomes. The FIRE-3 study [14] was conducted to evaluate the superiority of FOLFIRI plus cetuximab to FOLFIRI plus bevacizumab as a first-line treatment for mCRC patients with wild-type *KRAS*. In patients with only wild-type *RAS* tumors, the median OS was significantly longer with cetuximab (33.1 M versus 25.6 M, HR = 0.70, 95% CI = 0.53–0.92, *p* = 0.011), but the median PFS was almost identical (10.4 M versus 10.2 M, HR = 0.93, 95% CI = 0.74–1.17, *p* = 0.54). A similar pattern of outcomes was observed in the PEAK trial [43], primarily comparing FOLFOX plus either panitumumab or bevacizumab in patients with previously untreated, wild-type *KRAS* mCRC. In patients with all wild-type *RAS*, PFS was 13.0 M in the panitumumab arm and 9.5 M in the bevacizumab arm (HR = 0.65, 95% CI = 0.44–0.96, *p* = 0.029), and median OS was 41.3 M in the panitumumab arm and 28.9 M in the bevacizumab arm (HR = 0.63, 95% CI = 0.39–1.02, *p* = 0.058).

The phase III CALGB/SWOG 80405 trial [42] could not demonstrate a benefit for initial cetuximab versus bevacizumab in combination with either FOLFIRI or FOLFOX, even in patients with only wild-type *RAS* tumors. In patients with wild-type *RAS* tumors, there was no significant difference in OS and PFS between the cetuximab and bevacizumab in combination with chemotherapy. However, there was a higher response rate achieved in the cetuximab arm (68.6% versus 53.6%, *p* < 0.01). A meta-analysis of these randomized controlled trials supports a potential benefit of first-line EGFR inhibitors plus chemotherapy versus bevacizumab plus chemotherapy with respect to OS (HR = 0.8, 95% CI = 0.68–0.93, *p* = 0.004) and ORR (OR = 0.57, 95% CI = 0.42–0.76) in patients with wild-type *RAS* mCRC [44]. Another phase III trial currently in progress, PARADIGM [45], is comparing panitumumab versus bevacizumab in combination with FOLFOX.

## 6. Primary Tumor Location as a Prognostic and Predictive Biomarker in mCRC

Another topic of interest is the prognostic and predictive significance of primary tumor location in mCRC patients with wild-type *RAS* tumors. Genetic and phenotypic differences may be derived from pre-existing disparities between the left and right colon with respect to embryonic origin, blood supply, innervation, lymphatic drainage, and lumen environment. Associations between a particular marker and prognosis or response to chemotherapy have been found to be site-specific [46,47,48,49].

Recently, retrospective analyses were conducted in patients with wild-type *RAS* mCRC from two large phase III trials, CRYSTAL and FIRE-3, in which mCRC were classified as left-sided or right-sided [50]. In the CRYSTAL trial, patients with right-sided tumors in the FOLFIRI plus cetuximab treatment group had a significantly shorter PFS and OS compared with equivalent patients with left-sided tumors (PFS: 8.1 M versus 12.0 M, HR = 1.77, 95% CI = 1.08–2.91, *p* = 0.02; OS: 28.7 M versus 18.5 M, HR = 1.93, 95% CI = 1.24–2.99, *p* = 0.003). Furthermore, the addition of cetuximab to FOLFIRI in patients with left-sided tumors significantly improved PFS (12.0 M versus 8.9 M, HR = 0.50, 95% CI = 0.34–0.72, *p* < 0.001), OS (28.7 M versus 21.7 M, HR = 0.65, 95% CI = 0.50–0.86, *p* = 0.002), and ORR (72.5% versus 40.6%, OR = 3.99, 95% CI = 2.40–6.62, *p* < 0.001), as expected based on the overall population. In patients with right-sided tumors, however, the addition of cetuximab conferred more limited benefits on PFS (8.1 M versus 7.1 M, HR = 0.87, 95% CI = 0.47–1.62, *p* = 0.66), OS (18.5 M versus 15.0 M, HR = 1.8, 95% CI = 0.65–1.81, *p* = 0.76), and ORR (42.4% versus 33.3%, OR = 1.45, 95% CI = 0.58–3.64, *p* = 0.43).

Results consistent with these were obtained from the FIRE-3 trial. Patients with left-sided tumors who received FOLFIRI plus cetuximab had significantly longer OS than those receiving FOLFIRI plus bevacizumab (38.3 M versus 28.0 M, HR = 0.63, 95% CI = 0.48–0.85, *p* < 0.002), but with no significant differences in PFS or ORR. In contrast, among patients with right-sided tumors, there were no significant differences in OS, PFS, or ORR for FOLFIRI plus cetuximab versus FOLFIRI plus bevacizumab. Moreover, multivariable analyses revealed that, in both studies, there was a significant interaction between primary tumor location and treatment modality for OS of patients with wild-type *RAS* tumors.

These results confirm the prognostic value of primary tumor location in wild-type *RAS* populations, and further suggest an interaction between tumor location and response to treatment. Right-sided tumors do not significantly benefit from the addition of cetuximab, in contrast to a marked benefit for left-sided tumors [48,51]. This difference may be derived from the molecular/genetic background, and/or from embryological or epidemiological factors. For example, right-sided tumors are associated with *BRAF* mutation, DNA hypermethylation (the CpG island methylator phenotype, CIMP), and hypermutated consensus molecular subtypes [52]. Comprehensive molecular and genetic analysis of specimens derived from phase III trials is needed to identify biological differences between right- and left-sided colons.

## 7. Resistance Mechanism to Anti-EGFR Therapies

**Figure 1:**
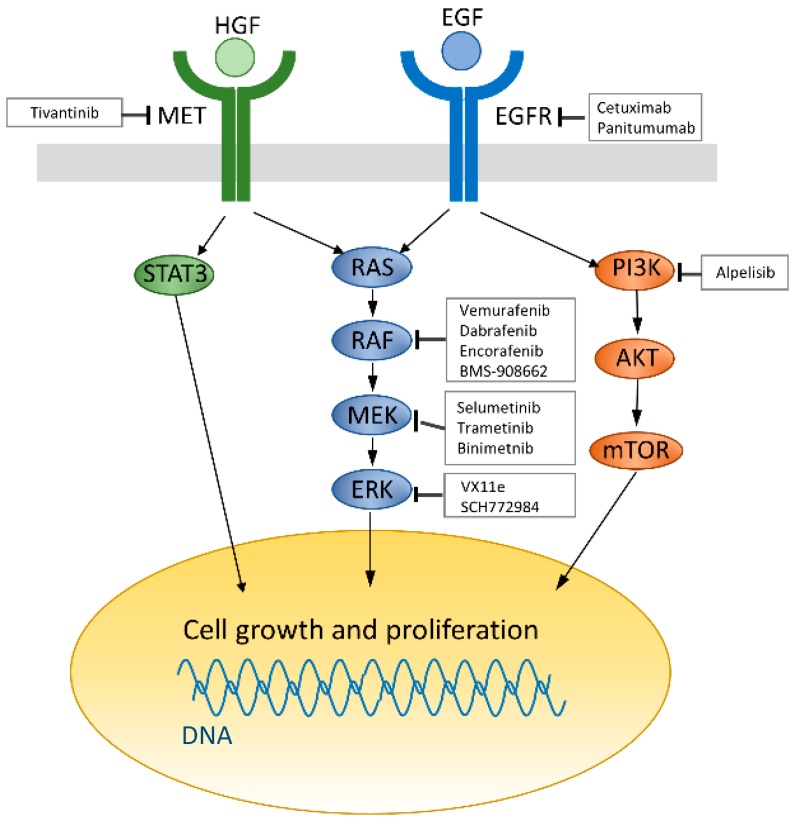
Current strategies for targeting the epidermal growth factor receptor (EGFR) pathway. EGF receptors (Blue) activate survival signaling pathways including RAS/MAPK and PI3K/AKT signaling pathway leading to cell growth and proliferation. HGF/MET (green) signaling also promotes cell growth and proliferation through RAS/MAPK and STAT3 phosphorylation. Regular arrow: activates, Arrow ending with a straight line: inhibits.

Even among patients with wild-type *RAS* mCRC that initially responds to anti-EGFR therapies, the majority will eventually experience disease progression. This implies acquired resistance to anti-EGFR therapy. Therefore, multiple studies have focused on exploring resistance mechanisms, and it seems that several biomarkers and pathways are involved in the development of resistance to anti-EGFR therapy. The two principal downstream effectors of EGFR activation are the RAS-MAPK pathway and the PI3K-AKT-mTOR pathway, both of which control cell growth and proliferation (Figure 1) [53]. Several investigators have assessed circulating tumor DNA (ctDNA) in the blood of mCRC patients during anti-EGFR therapy and found that undetectable low-frequency *KRAS*-mutant clones may be selected by anti-EGFR treatment [54,55,56,57].

Using BEAMing technology, which can detect somatic mutations in small amounts of ctDNA, Morelli et al. found that acquired mutations in *KRAS* were seen in 44% of patients initially characterized as having wild-type *KRAS* tumors, following the development of resistance to anti-EGFR therapy [54]. Interestingly, atypical *KRAS* codon 61 and 146 mutations were more frequent among these newly diagnosed mutations. In addition, the mutations were detectable as low-allele-frequency clones in 35% of patients with plasma mutations, suggesting that they were already present in the treatment-naïve primary tumors. The presence of low-frequency *KRAS* mutations in these patients was correlated with a poorer response to anti-EGFR treatment. These data suggest that a high-sensitivity mutation detection technique that provides quantitative information about the presence of mutant alleles in the gene of interest would allow for the selection of patients most likely to benefit from anti-EGFR treatment.

A second mechanism of resistance entails mutations in the extracellular domain (ECD) of *EGFR* that prevent binding of the drug to the receptor. Although secondary *EGFR* mutation is rarely detected after resistance to anti-EGFR therapies, Montagut and colleagues discovered an acquired *EGFR* mutation, S492R, and proved its association with acquired resistance to cetuximab in mCRC [58]. The S492R mutation is within the EGFR ectodomain, and a bulky side chain at this position could interfere with cetuximab binding. The *EGFR^S492R^* mutation was detected in two of ten patients who experienced disease progression after a prior response to cetuximab with chemotherapy. Interestingly, one patient with cetuximab resistance harboring the *EGFR^S492R^* mutation responded to treatment with panitumumab. This result was also confirmed in vitro by using NIH3T3 fibroblasts expressing wild-type or S492R-mutant *EGFR*. In cells carrying the S492R mutation, only panitumumab blocked the activation of EGFR. Overall, these findings identified the S492R mutation as a mechanism of acquired but not primary resistance to cetuximab.

Some new EGFR inhibitors appear to overcome resistance to cetuximab or panitumumab that is due to the emergence of mutations in the *EGFR* ECD. Sym004 is a novel 1:1 mixture of two non-overlapping anti-EGFR monoclonal antibodies that target different epitopes of the *EGFR* ECD. A unique feature of Sym004 is its ability to mediate rapid EGFR internalization and subsequent degradation via EGFR cross-linking [59,60,61]. Preclinical studies with Sym004 showed superior antitumor activity as compared with cetuximab or panitumumab, as well as activity in models of acquired cetuximab resistance [60,62]. In a phase I study of mCRC patients whose tumors were resistant to cetuximab or panitumumab, Sym004 demonstrated a 44% tumor shrinkage rate and a 13% response rate [59]. It is currently being tested in a dose-optimization, phase II study in refractory mCRC patients (NCT02083653), as well as in combination with FOLFIRI (NCT02568046) [63].

MM-151 is a combination of three fully human IgG1 monoclonal antibodies that can simultaneously engage distinct, non-overlapping epitopes on the EGFR [64,65]. MM-151 was designed using a systems biology approach to substantially improve on the mechanisms that enable potent EGFR antagonism, *EGFR* downregulation, and immune effector function. In a phase I study examining MM-151 as a monotherapy or in combination with irinotecan, three mCRC patients achieved partial responses, and eight had stable disease for more than 4 M on single-agent MM-151 [66]. The most common adverse events were toxicities related to the EGFR pathway, including rashes (70%), hypomagnesemia (24%), fatigue, dry skin, and diarrhea (21%). After MM-151 treatment, liquid biopsies of patients with *EGFR* ECD mutations at baseline showed decreased or stabilized *EGFR* ECD mutant DNA concentrations that paralleled response assessments using radiological methods.

Activation of the PI3K/AKT/mTOR signaling pathway has also been implicated as an important mechanism in the resistance to EGFR inhibitors. *PIK3CA* mutations occur at a prevalence of 10%–20%, are mainly located at hot spots in exons 20 and 9, and constitutively activate downstream signaling [67]. Retrospective studies have investigated the potential role of *PIK3CA* mutations as predictors of resistance to anti-EGFR treatment in mCRC patients, with conflicting results. A systematic review was conducted to evaluate the association between *PIK3CA* mutations and resistance to anti-EGFR therapy in mCRC [68]. The results showed a trend of worse PFS and a significantly shorter OS (HR = 1.43, 95% CI = 1.02–2.00) compared with patients with wild-type tumors. However, a large prospective study is needed to confirm this result.

Additional possible mechanisms of resistance to anti-EGFR therapies have been proposed in patients with *RAS* and *BRAF* wild-type mCRC [69]. Genetic aberrations of the receptor tyrosine kinases *c-MET* and *ERBB2* are identified as the bypass mechanisms for acquired resistance to anti-EGFR therapies. c-MET and its ligand HGF have been previously implicated in both primary and acquired resistance to anti-EGFR therapies. For example, among 103 patients with *KRAS* wild-type mCRC who received anti-EGFR therapies, those with high serum levels of HGF had shorter PFS (4.4 M versus 6.4 M, *p* = 0.001) and OS (8.0 M versus 15.3 months; *p* = 0.001) compared with those with low levels of HGF [70]. Another study demonstrated that c-MET overexpression significantly correlated with shorter PFS (3 M versus 5 M, *p* = 0.018) and OS (11 M versus 10 M, *p* = 0.037) compared with low/normal expression [71]. Whole-exome analysis revealed that the c-MET copy number was increased in CRC tissues from patients who developed resistance to anti-EGFR therapies [72]. Moreover, the c-MET amplification locus could be detected in circulating tumor DNA, suggesting that the early initiation of c-MET inhibitors in those patients who respond to anti-EGFR therapies and do not display emergence of *KRAS* mutations in blood tests during anti-EGFR therapies [72].

ERBB2 gene amplifications were also described as drivers of acquired resistance to anti-EGFR therapies in *RAS* and *BRAF* wild-type mCRC. Yonesaka et al. showed that activation of HER2 signaling in cancer cells, through either *HER2* gene amplification or upregulation of the HER2/HER3 ligand heregulin, is enabled to acquire the resistance to anti-EGFR therapies [73]. Large xenograft cohorts from patient-derived demonstrated that ErbB-2 amplification was shown in cetuximab-resistant patients and in *RAS/BRAF/PI3KCA* wild-type patients [74]. Moreover, HER2 amplification was detected in ctDNA from patients with mCRC and acquired resistance to anti-EGFR antibody therapy [75,76]. These data suggest that combination therapies against EGFR and ERBB2 in *RAS* wild-type tumors might be useful.

## 8. BRAF Mutation in Colorectal Cancer

*BRAF* is a serine/threonine protein kinase that plays an important role in the *EGFR*-mediated *MAPK* pathway [77], where it is activated by the small GTPase, *RAS*. The *BRAF* gene is somatically mutated in approximately 5% to 10% of mCRC [78,79]. The most common *BRAF* mutation in CRC is the V600E alteration, which causes a constitutive activation of the MAPK pathway, independent of RAS activity [80]. In addition, *BRAF* mutations are mutually exclusive with *RAS* mutations [81]. The *BRAF^V600E^* mutation is associated with proximal location, greater age, female gender, microsatellite instability (MSI), CIMP, high grade, and mucinous histology in colorectal cancer [81]. *BRAF* mutations have also been shown to be associated with poor prognosis in mCRC. Moreover, *BRAF* mutations also appear to have predictive value, with increasing evidence that *BRAF* mutations predict a lack of benefit from anti-EGFR therapy, even in wild-type *RAS* mCRC. Currently, consensus-based guidelines from NCCN [18] and ESMO [19] both recommend avoiding the use of cetuximab or panitumumab for patients with *BRAF*-mutated cancers.

## 9. Combination Therapies with BRAF Inhibitors

*BRAF* mutations are commonly identified in melanoma, in which the *BRAF* mutation rate is more than 60%, offering hope that inhibition of BRAF kinase activity could benefit melanoma patients. The BRAF inhibitors, vemurafenib and dabrafenib, have shown response rates of up to approximately 50% in metastatic melanoma patients with the *BRAF^V600E^* mutation [82,83]. However, BRAF inhibitor monotherapies appear to be ineffective in *BRAF^V600E^* mutant mCRC, with a response rate of approximately 5% [79]. Accumulating evidence suggests that these BRAF inhibitors induce negative feedback reactivation of EGFR, which in turn activates MAPK via CRAF and RAS [84,85,86].

Preclinical studies indicated that combinations of BRAF inhibitors with inhibitors of upstream receptors show an improved efficacy in *BRAF*-mutated mCRC [87]. Based on these data, BRAF inhibitor combinations have been evaluated in clinical trials for patients with *BRAF*-mutated mCRC in recent years, and are showing signs of improved efficacy compared with RAF inhibition alone (Table 1) [85,88,89,90]. Kopetz and colleagues presented the results of a phase II/III trial in which they randomly assigned 49 patients with *BRAF*-mutated mCRC to receive irinotecan, cetuximab, and vemurafenib and 50 to receive only irinotecan and cetuximab [91]. Patients receiving the three-drug regimen showed significantly longer PFS compared with those receiving the doublet regimen (4.4 M versus 2.0 M, HR = 0.42, 95% CI = 0.26–0.66, *p* = 0.0002). Notably, Grade 3 or 4 toxicities in patients in the three-drug group included neutropenia (28%), diarrhea (22%), anemia (13%), and nausea (15%). However, these are similar to a prior second line study of cetuximab plus irinotecan. These results for combination therapy including a BRAF inhibitor are consistent with earlier preclinical studies.

Despite combination therapy initially preventing the development of acquired resistance in patients with *BRAF*-mutated mCRC, these patients ultimately develop secondary resistance to treatment and experience disease progression. Alterations in MAPK pathway genes, including *KRAS* amplification, *BRAF* amplification, and a *MEK1* mutation, were found in resistant tumors and were not present in matched pretreatment tumors [92]. Importantly, an ERK inhibitor retained the ability to suppress the MAPK pathway and could overcome each of the identified acquired resistance mechanisms in *BRAF*-mutated mCRC [92]. These results suggest that ERK inhibitors could be important components of future therapeutic strategies for *BRAF*-mutated mCRC, either alone or in combination with BRAF and EGFR inhibitors.

## 10. Conclusions

EGFR-targeting drugs directly affect cancer cells and may be affected by accumulating drug resistance mutations in the RAS-RAF-MEK-ERK pathway. Several clinical trials are currently underway to elucidate which treatment has the best outcome. We believe that liquid biopsy and next-generation sequencing technology will help to identify and monitor predictive biomarkers for acquired resistance to anti-EGFR therapies. One can hope that a comprehensive understanding of resistance mechanisms will contribute to developing more effective strategies to overcome acquired resistance in the future.

## Figures and Tables

**Table 1:** Clinical trials of drugs targeting EGFR downstream pathway.

Strategy	Treatment	Genomic Profile	Phase	N	ORR (%)	PFS (M)	OS (M)	Clinical Development	Reference
BRAF monotherapy	Vemurafenib	BRAFmut	I/II	21	5	2.1	7.7	Complete	NCT00405587 [93]
BRAF monotherapy	Vemurafenib	BRAFmut	II	10	0	4.5	9.3	Complete	NCT01524978 [94]
BRAF + MEK	Dabrafenib + Trametinib	BRAFmut	I/II	43	12	3.5	(−)	Complete	NCT01726738 [88]
BRAF + EGFR	Vemurafenib + Cetuximab	BRAFmut	II	27	4	3.7	7.1	Complete	NCT01524978 [94]
BRAF + EGFR	Vemurafenib + Panitumumab	BRAFmut	Pilot	15	13	3.2	7.6	Complete	NCT01791309 [89]
BRAF + EGFR	Encorafenib + Cetuximab	BRAFmut	II	50	22	4.2	12.4	Ongoing	NCT01719380 [90]
BRAF + EGFR	Dabrafenib + Panitumumab	BRAFmut	I/II	20	10	3.4	(−)	Ongoing	NCT01750918 [85]
BRAF + EGFR	BMS-908662 + Cetuximab	Kras mut or BRAF mut	I/II	17 *	(−)	(−)	(−)	Ongoing	NCT01086267
BRAF + EGFR + CT	Vemurafenib + Cetuximab + irinotecan	BRAFmut	I	12	50	(−)	(−)	Ongoing	NCT01787500
BRAF + EGFR + CT	Cetuximab + irinotecan ± Vemurafenib	BRAFmut	II (RCT)	78 *	(−)	(−)	(−)	Ongoing	NCT02164916
BRAF + EGFR + MEK	Dabrafenib + Panitumumab + Trametinib	BRAFmut	I/II	83	18	(−)	(−)	Ongoing	NCT01750918 [85]
BRAF + EGFR ± MEK	Encorafenib + Cetuximab ± Binimetinib	None	III	645 *	(−)	(−)	(−)	Ongoing	NCT02928224
BRAF + EGFR + PI3K	Encorafenib + Cetuximab + Alpelisib	BRAFmut	II	52	27	5.4	13.1	Ongoing	NCT01719380 [90]
MEK + CT	Selumetinib + irinotecan	Kras mut or BRAF mut	II	32	9	(−)	(−)	Complete	NCT01116271 [95]
MEK + EGFR	Binimetinib + Panitumumab	RAS wt or RAS mut	I/II	90 *	(−)	(−)	(−)	Ongoing	NCT01927341
MEK + EGFR	Panitumumab + Trametinib	BRAFmut	I/II	31	0	2.8		Ongoing	NCT01750918 [85]
MEK + EGFR	Trametinib + Panitumumab	KRAS BRAF wt	II	26 *	(−)	(−)	(−)	Ongoing	NCT02399943
MET + EGFR + CT	Cetuximab + irinotecan ± Tivantinib	KRAS wt	I/II (RCT)	60	45	8.3	19.8	Complete	NCT01075048 [96]
MEK + AKT	Selumetinib + MK-2206	KRAS wt or KRAS mut	II	21 *	(−)	(−)	(−)	Ongoing	NCT01333475

ORR: objective response rate; PFS: progression free survival; OS: overall survival; M: months; CT: chemotherapy; RCT: randomized control trial. * Planed sample size, (-): not reported.
